# Determination of Testicular Blood Flow in Camelids Using Vascular Casting and Color Pulsed-Wave Doppler Ultrasonography

**DOI:** 10.4061/2011/638602

**Published:** 2011-09-19

**Authors:** Michelle Kutzler, Reid Tyson, Monica Grimes, Karen Timm

**Affiliations:** ^1^Department of Animal Sciences, College of Agricultural Sciences, Oregon State University, Corvallis, OR 97331, USA; ^2^Department of Small Animal Clinical Sciences, VA-MD Regional College of Veterinary Medicine, Virginia Tech, Blacksburg, VA 24061, USA; ^3^College of Veterinary Medicine, Oregon State University, Corvallis, OR 97331, USA

## Abstract

We describe the vasculature of the camelid testis using plastic casting. We also use color pulsed-wave Doppler ultrasonography to measure testicular blood flow and compare the differences between testicular blood flow in fertile and infertile camelids. The testicular artery originates from the ventral surface of the aorta, gives rise to an epididymal branch, and becomes very tortuous as it approaches the testis. Within the supratesticular arteries, peak systolic velocity (PSV) was higher in fertile males compared to infertile males (*P* = 0.0004). In addition, end diastolic velocity (EDV) within the supratesticular arteries was higher for fertile males when compared to infertile males (*P* = 0.0325). Within the marginal arteries, PSV was also higher in fertile males compared to infertile males (*P* = 0.0104). However, EDV within the marginal arteries was not significantly different between fertile and infertile males (*P* = 0.121). In addition, the resistance index was not significantly different between fertile and infertile males within the supratesticular (*P* = 0.486) and marginal arteries (*P* = 0.144). The significance of this research is that in addition to information obtained from a complete reproductive evaluation, a male camelid's fertility can be determined using testicular blood flow measured by Doppler ultrasonography.

## 1. Introduction

Over the last three decades, domesticated llamas (*Lama glama*) and alpacas (*Vicugna pacos*) have become increasingly popular in the United States, Australia, and Europe. As a result, they are kept in very different climatic regions than where they are native (the Andean mountain range of South America). High humidity and environmental temperatures cause heat stress and infertility in male camelids, possibly because of scrotal anatomical differences in these species compared to other mammals. In camelids, the scrotum is located in the perineum (situated just ventral to the ischial arch) and closely positioned next to the body wall [1]. Also, the skin of the scrotum is thick, presumably an adaptation to prevent against injury during male-male fighting [2]. 

As in most domestic species, camelid breeders strive to improve the health, production, and breed characteristics of their animals through carefully monitored breeding programs. Unfortunately, there are several aspects of camelid reproductive physiology that make breeding camelids especially difficult. When compared to other domestic species, camelids have poor fertility and pregnancy rates. For instance, only 40–60% of natural matings result in offspring [3]. 

Techniques used to diagnose infertility in llamas are challenging. While a semen evaluation is a standard practice prior to a sale or when infertility is suspected in other species, semen evaluation is rarely done in camelids because of difficulties with semen collection and analysis. In camelids, ejaculation is a continuous process that occurs throughout copulation lasting 5–50 minutes in a sternal recumbent position [1, 4, 5]. Intravaginal sacs and condoms [5, 6] were the first described methods for semen collection in camelids. However in many cases, samples collected using these methods contain no sperm (aspermic) because the male could not penetrate the penis within the cervix, which is a trigger for ejaculation [5, 6]. Semen collected by electroejaculation is often contaminated with urine and does not yield a reliable or representative semen sample [6]. Because of these problems, there is a need to identify alternative diagnostic tools for infertility in camelids.

Information on reproductive abnormalities, specifically those pertaining to spermatogenesis, in camelids is extremely limited. Many male camelids have a high percentage of spermatozoa with abnormal morphology. These abnormalities include variable sperm head sizes and sperm head and midpiece vacuolar defects [7]. In cattle, swine, and humans, it has been shown that such morphologic abnormalities, in addition to genetic reasons, can result from poor scrotal thermoregulation or decreased testicular blood flow [8]. This may also be true for camelids. 

Color pulsed-wave Doppler ultrasound has become a method of choice to evaluate vasculature of various organs, including testes. In human medicine, this technique has been employed to evaluate blood flow in the testicular artery and has been applied in diagnosing testicular pathologies associated with altered blood flow [9–12]. Literature pertaining to the camelid testicular vasculature is very sparse. There has been no research that characterizes the vascular bed within the camelid scrotum or normal blood flow to the testes. Furthermore, there have been no studies on real-time testicular blood flow determination in camelids. 

Determining testicular blood flow in normal camelids will assist in the etiologic diagnosis of abnormal spermatozoal morphology as well as provide a standardized method to use during breeding soundness evaluations. The objectives of this project were to (1) describe the vasculature of the camelid testis and identify candidate arteries that can be used to determine testicular blood flow using color pulsed-wave Doppler ultrasonography; (2) determine the blood flow to and within the testes in normal camelids and in camelids with a reproductive history infertility; (3) compare the differences in testicular blood flow between fertile and infertile camelids. The significance of this research is that measuring testicular blood flow will assist in the etiologic diagnosis of abnormal sperm morphology in camelids as well as provide a standardized method to use during breeding soundness evaluations.

## 2. Materials and Methods

### 2.1. Vascular Casting

A fertile six-year-old male alpaca was used to produce a vascular cast of the terminal testicular vasculature. This male had sired many offspring and had recently produced pregnancies. The left external jugular vein was catheterized. The alpaca was heparinized (40,000 IU sodium heparin IV) and anesthetized with intravenous xylazine (0.66 mg/kg) and ketamine (0.5 mg/kg) in one liter of 5% guaifenesin. The alpaca was then placed in dorsal recumbency, and a ventral midline laparotomy was performed. The abdominal aorta and caudal vena cava were cannulated near the origin of testicular artery and insertion of the testicular vein, respectively. The alpaca was then euthanized under anesthesia by exsanguinations, and sixty liters of heparinized saline was perfused through the aorta and out of the caudal vena cava at a pressure of 100 mm Hg using a peristaltic pump. Batson's no. 17 casting resin (Batson's no. 17 Plastic Replica and Corrosion Kit; catalog number 07349; Polysciences, Inc.; Warrington, Pa) was freshly prepared according to the user manual in a cold beaker using the proportions 10 : 1 : 0.1 for base monomer, solution B Batson's no. 17 catalyst and solution C promoter, respectively. A small amount (50 mg) of dye was added (red for arterial and blue for venous) for visualization. The resin was injected through the abdominal aorta and caudal vena cava cannulas using a peristaltic pump to determine position and size of the major vessels entering and leaving the testes. 

Complete polymerization of the resin (curing) within the tissue took 72 hours at 4°C. Polymerization at the lower temperature was used to minimize distortion of the vascular cast during the exothermic reaction. The pelvic region was dissected away from the cadaver and then placed into a 6% potassium hydroxide solution for 72 hours at room temperature to remove any remaining tissue. The solution corroded the tissues away from the polymerized plastic within the vascular tree lumens. Castings were washed with water several times, allowed to dry, and then photographed. This method of vascular casting was first described 55 years ago [13] and has since been validated for characterizing the intricate vessels within the hoof in horses [14], skin in rabbits [15], kidneys in rats [16], and gills in pufferfish [17]. 

In addition, a virgin two-year-old male llama was routinely castrated, and the testicular artery from each of the castrated testes was cannulated. Sixty milliliters of heparinized saline was perfused through the testicular artery and out of the pampiniform plexus (testicular vein). The testicular artery from each castrated testes was perfused as previously described to cast the arterial vasculature within the testes. The resin was allowed to cure for 24 hours at 4°C and then the testes were placed into a 3% potassium hydroxide solution for 72 hours at room temperature to remove any remaining tissue. Castings were washed with water several times, allowed to dry, and then photographed.

### 2.2. Color Pulsed-Wave Doppler Ultrasonography

Fourteen camelids (7 fertile and 7 infertile) were used in this study. In addition to obtaining a reproductive history and performing, a complete physical examination, age (years), body weight (kg), and testicular volume (cm^3^) were recorded (Table 1). The camelids were then sedated with butorphanol (15 mg IV and 15 mg IM), blindfolded with a towel, and positioned in sternal recumbency. A Philips iU22 ultrasound system with an L12-5 probe was used. The skin of the scrotum was cleaned, and alcohol and contact gel were applied to the scrotal surface to facilitate ultrasonographic imaging. Color pulsed-wave Doppler ultrasonography was used to measure peak systolic velocity (PSV), end diastolic velocity (EDV), and resistance index (RI) in vessels identified from the casting study (Table 2). Testicular arteries from two locations were evaluated: (1) cranioventral to the caput epididymis (supratesticular artery) and (2) caudal to long axis of testis (epididymal edge of marginal artery). In most cases, the vessels were imaged in sagittal or oblique sagittal planes. The largest observable section of each artery was identified using real-time scans. Point spectral analysis was performed on the largest vessel identified for each category using pulsed Doppler scans. Suitability for Doppler analysis was determined on the basis of whether vessels had a discernible course, determinable axis, and adequate flow intensity. The Doppler gate of 4 ± 7 mm length was placed within the vessel. The angle between the Doppler beam and the long axis to the vessel was less than 60 degrees. To observe slow blood flows, the color gain was adjusted to reduce excessive color noise. The duration of the ultrasound examination did not exceed 30 minutes.

### 2.3. Data Analysis

**Figure 1:**
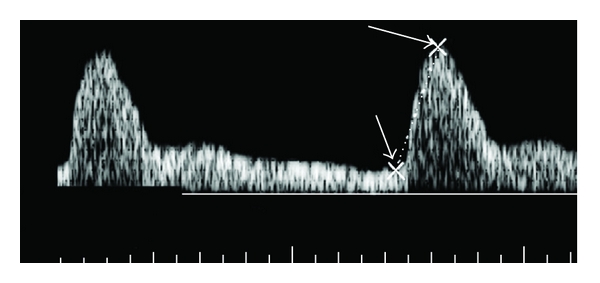
Pulsed-waveform of a supratesticular artery illustrating measurement of peak systolic velocity (right arrow) and end diastolic velocity (left arrow).

For each spectrum, the first and last complete cardiac cycles were measured, and the PSV, ESV, and RI were determined. For the calculation of time-averaged mean velocity of a cardiac cycle, the spectral curve of the cardiac cycle was marked manually with a cursor (Figure 1). The cursor indicates the area beneath the curve to determine the flow velocity interval, which can be divided by the duration of the cardiac cycle to yield the mean velocity. Three sweeps were averaged to obtain a single mean value for each measure at each location. Left and right PSV, EDV, and RI for supratesticular and marginal arteries were averaged for each individual. Mean ± SEM for each group was determined, and comparison of the Doppler results with fertility status was made using a one-way unpaired Student's *t*-test. Significance was defined as *P* < 0.05.

## 3. Results

**Figure 2:**
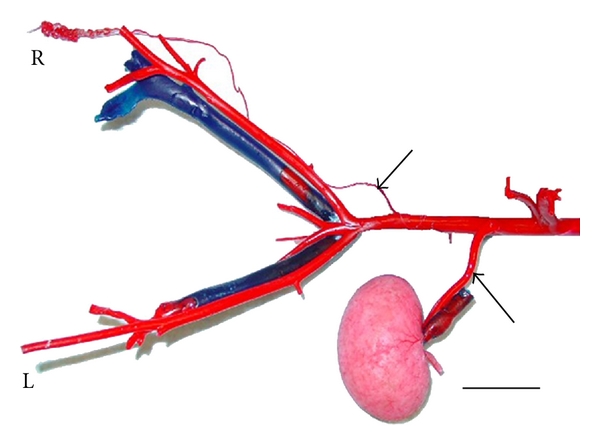
Vascular casting of caudal abdominal aorta from a fertile six-year-old male alpaca. This male had sired many offspring and had recently produced pregnancies. Under general anesthesia, the abdominal aorta and caudal vena cava were cannulated near the origin of testicular artery and insertion of the testicular vein, respectively. The alpaca was then euthanized under anesthesia by exsanguination and Batson's no. 17 casting resin (red for arterial and blue for venous) injected through the abdominal aorta and caudal vena cava cannulas using a peristaltic pump. The pelvic region was dissected away from the cadaver and then placed into a 6% potassium hydroxide solution for 72 hours at room temperature to remove any remaining tissue. The left (L) renal artery (bottom arrow), right (R) testicular artery (top arrow), and the termination of the abdominal aorta are shown. Bar = 5 cm.

**Figure 3:**
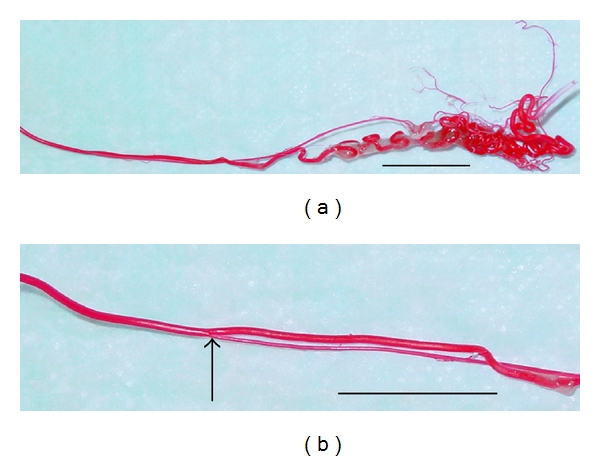
Vascular casting of the right testicular artery (a) as it enters the testis from the same male used in Figure 2. An epididymal branch (arrow) arises from the testicular artery (b). Bars = 2 cm.

**Figure 4:**
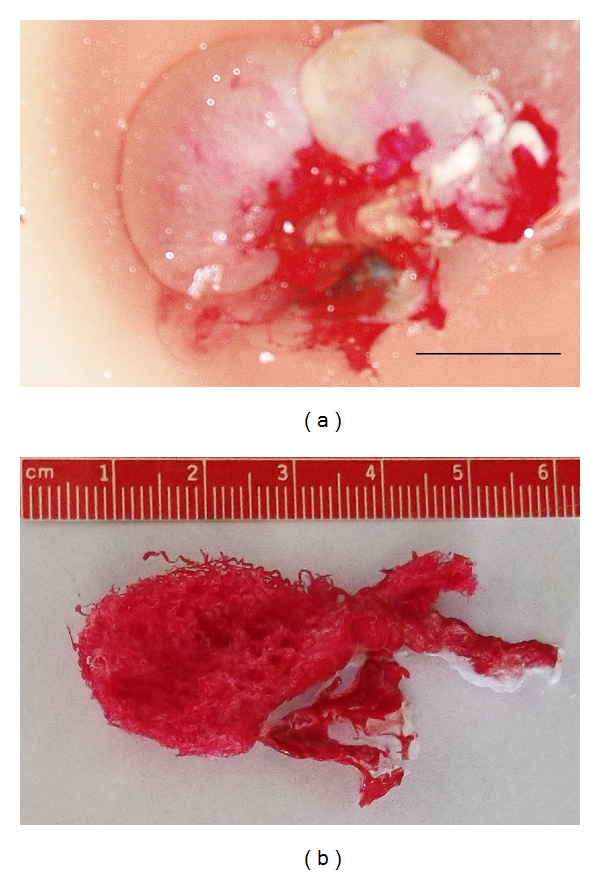
Vascular casting of the right testicular artery within the testis from a virgin two-year-old male llama. This male was routinely castrated, and the testicular artery from the right castrated testes was cannulated. Sixty milliliters of heparinized saline was perfused through the testicular artery and out of the pampiniform plexus (testicular vein). The right testis was perfused with Batson's no. 17 casting resin as described in Figure 2 to cast the arterial vasculature within the testes. The testis was placed into a 3% potassium hydroxide solution for 72 hours at room temperature to remove any remaining tissue. (a) Illustrates the technique for casting a castrated testis (Bar = 2 cm); (b) Illustrates the vascular cast produced from the right testicular artery within the testis illustrating the small vessels (recurrent rami) within the testis. Pulsatility could not be consistently measured with these vessels for use in the comparison of fertile and infertile camelids.

The caudal vena cava leaked during perfusion, and, subsequently, only larger veins (e.g., external iliacs) were preserved. The testicular veins were not cast. The left renal artery, right testicular artery, and the termination of the abdominal aorta were well cast (Figure 2). The right renal artery was damaged during catheterization of the aorta. The left testicular artery did not perfuse with casting material. The testicular artery originates from the ventral surface of the aorta, gives rise to an epididymal branch, and becomes very tortuous (supratesticular artery) as it approaches the testis (Figure 3). The terminating branches of the testicular artery distal to the convoluted section were not preserved on this specimen. From the castrated testes, a portion of the marginal artery was perfused in addition to several small intratesticular arterioles (recurrent rami) (Figure 4). These arteries were named based on anatomic similarities to vessels identified in dogs, horses, and humans [18–20].

All of the animals used in this study were in good physical health. Fertility status (fertile versus infertile) was determined based upon owners' responses to questions regarding the male reproductive history. Males defined as fertile for this study had been bred to numerous females and produced pregnancies within six months prior to their examination for this study. Males defined as infertile for this study had failed to produce a pregnancy within the preceding six months despite numerous mating attempts to multiple females that had known previous and subsequent fertility as demonstrated by becoming pregnant and carrying a fetus to term. There were no significant differences between fertile and infertile males in age (6.42 ± 4.54 years and 4.28 ± 2.16 years, resp.; *P* = 0.141), body weight (140.71 ± 8.27 kg and 124.74 ± 18.05 kg, resp.; *P* = 0.058), or testicular volume (11.76 ± 2.79 cm^3^ and 11.14 ± 2.89 cm^3^, resp.; *P* = 0.342) (Table 1).

**Figure 5:**
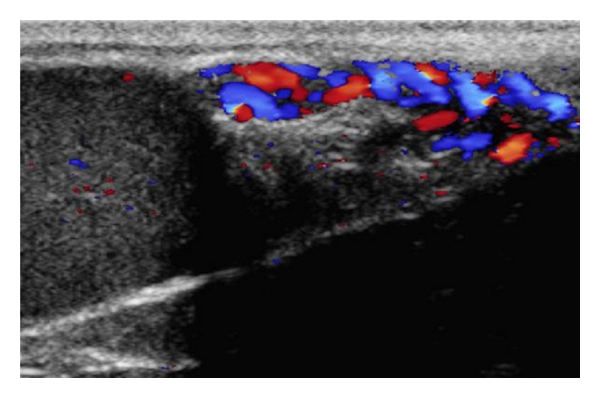
Ultrasonogram of a testis using color pulsed-wave Doppler ultrasonography showing blood flow within the supratesticular artery.

**Figure 6:**
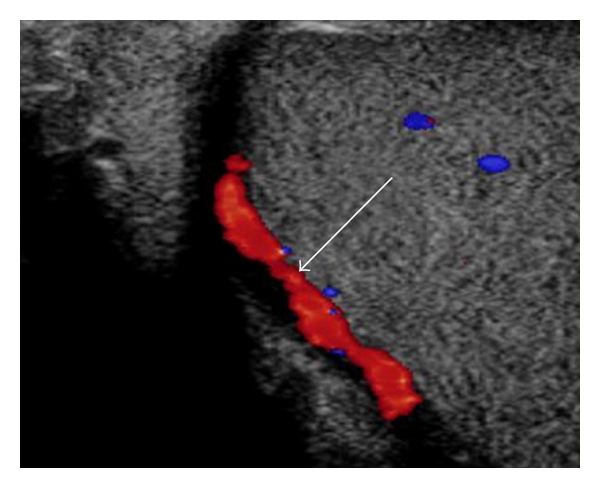
Ultrasonogram of a testis using color pulsed-wave Doppler ultrasonography showing blood flow within the marginal artery (arrow).

**Figure 7:**
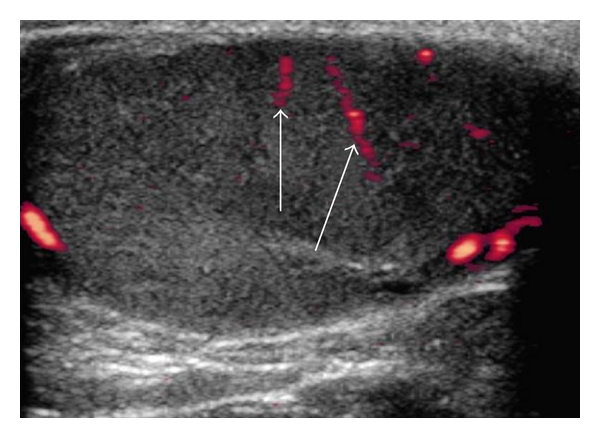
Ultrasonogram of a testis using color pulsed-wave Doppler ultrasonography showing blood flow within the recurrent rami (arrows).

The blood flow of the supratesticular artery (Figure 5) and the marginal artery (Figure 6) was determined by pulsed-wave Doppler ultrasonography (Table 2). There were no significant differences between the left and right sides (*P* > 0.55). The epididymal arteries could not be detected. Color flow within the recurrent rami was visible as small red or blue dots scattered throughout the parenchyma (Figure 7). However, these intratesticular arteries/arterioles were too small to consistently obtain pulsed-wave Doppler measures of blood flow. Using pulsed-wave Doppler ultrasonography, there were no vascular alterations (e.g., varicoceles, varicosities) visualized within the testicular vasculature of the animals studied. 

Within the supratesticular arteries, the mean ± SEM for PSV was higher in fertile males compared to infertile males (21.41 ± 1.11 cm/s versus 15.09 ± 1.09 cm/s, resp.; *P* = 0.0004). In addition, the mean ± SEM for EDV within the supratesticular arteries was higher for fertile males when compared to infertile males (6.51 ± 0.81 cm/s versus 4.46 ± 0.41 cm/s, resp.; *P* = 0.0325). However, the mean ± SEM for RI within the supratesticular arteries was not significantly different for fertile males when compared to infertile males (0.69 ± 0.09 versus 0.69 ± 0.08 cm/s, resp.; *P* = 0.486). Within the marginal arteries, the mean ± SEM for PSV was also higher in fertile males compared to infertile males (13.18 ± 0.93 cm/s and 10.17 ± 0.55 cm/s, resp.; *P* = 0.0104). However, the mean ± SEM for EDV within the marginal arteries was not significantly different between fertile and infertile males (7.29 ± 0.57 cm/s and 6.18 ± 0.40 cm/s, resp.; *P* = 0.121). In addition, the mean ± SEM for RI within the marginal arteries was not significantly different for fertile males when compared to infertile males (0.45 ± 0.08 versus 0.39 ± 0.09 cm/s, resp.; *P* = 0.144).

## 4. Discussion

The results of this study show that color pulsed-wave Doppler ultrasonography is a useful noninvasive method to measure the blood flow of the testicular arteries in male camelids. Similar results have been reported in dogs [18], horses [19], and man [20, 21]. Just as in stallions [22], there were no significant differences between the left and right camelid testes in any of the blood flow parameters measured. In stallions, the epididymal arteries arising from the testicular artery after entering the testis [23]. While in contrast, the dog has two epididymal branches (cranial and caudal) arise from the testicular artery prior to entering the testis [24]. In the current study, the epididymal branch of the camelid testicular artery also arises prior to entering the testis. However, unlike in the dog, only one branch was identified in the specimen presented here. Similar to reports in dogs [18], horses [19], and man [20, 21], epididymal arterial blood flow could not be detected using color pulsed-wave Doppler ultrasonography. 

As it has previously been reported in horses [25], irrespective of the individual examined, pulsatile flow could be repeatedly measured in both the supratesticular and marginal arteries. However, pulsatile flow within the recurrent rami (intratesticular vessels) was inconsistent between and within individuals. This may have resulted from a flow velocity below the ultrasound unit's sensitivity threshold. From the two locations of the testicular artery examined in the present study, the supratesticular artery proved to be the more useful for supporting the diagnosis of infertility because both the PSV and EDV differed between fertile and infertile males. 

The supratesticular artery is closely associated with veins of the pampiniform plexus, which is often referred to as the “vascular cone” [26, 27]. In bulls, the cone-shaped pampiniform plexus encases the spiral-shaped supratesticular artery to cool blood before entering the testes [28, 29]. In man, the supratesticular artery is straighter, and, in 75% of cases, the human artery did not form the loops which were present in 100% of the bovine specimens [30]. In the present study, the camelid supratesticular artery is similar to that of the bull, which is very tortuous as it approaches the testis.

The vascular cone is a countercurrent heat transfer system, such that heat is transferred from the warm blood in the testicular artery to the cooler blood in the testicular venous system. This thermoregulatory mechanism maintains the testis parenchyma 4-5° below core body temperature. Several investigations in humans have demonstrated an association between testicular blood flow and sperm quality [31–34] and rats [35, 36]. Any process which decreases blood flow will alter these thermoregulatory mechanisms and can result in infertility. In the current study, we found that male camelids with a history of infertility also had lower testicular blood flow.

## 5. Conclusion

The importance and relevance of this work is that it is the first report to describe the vasculature of the camelid testes and can be employed as a guide to identify candidate arteries that can assist in determining testicular blood flow in camelids. Then, we (1) identified the major blood vessels supporting the llama testes using color pulsed-wave Doppler ultrasonography; (2) determined the blood flow to and within the testes in normal llamas and in llamas with a reproductive history of infertility; (3) compared the differences in testicular blood flow between fertile and infertile llamas. The significance of this research is that in addition to information obtained from a complete reproductive evaluation, a male camelid's fertility can be determined using testicular blood flow measured by Doppler ultrasonography.

## Figures and Tables

**Table 1:** Animal information gathered regarding fertility status, age (years), body weight (kg), and testicular volume (cm^3^) for fourteen camelids used in this study.

Species	Fertility	Age (years)	Weight (kg)	Volume (cm^3^)	
				Left testis	Right testis
Llama	Fertile	13	131.82	16.26	16.47
Llama	Fertile	3	137.27	18.20	9.40
Llama	Fertile	3	150.00	11.66	12.09
Llama	Fertile	3	129.55	10.05	10.80
Llama	Fertile	12	150.00	10.65	9.85
Llama	Fertile	8	145.45	15.40	8.66
Llama	Fertile	3	140.91	7.42	7.78
Llama	Infertile	6	152.27	11.83	13.50
Llama	Infertile	2	121.36	9.80	4.30
Llama	Infertile	5	105.45	18.68	12.42
Llama	Infertile	2	121.36	12.62	8.19
Llama	Infertile	6	147.73	11.20	9.91
Llama	Infertile	7	138.64	14.63	11.26
Alpaca	Infertile	2	86.36	12.48	5.08

**Table 2:** Peak systolic velocity (PSV), end diastolic velocity (EDV), and resistance index (RI) for supratesticular and marginal arteries pulsatility from the left (L) and right (R) testes from fourteen camelids as determined using color pulsed-wave Doppler ultrasonography.

Species	Fertility status	Supratesticular artery						Marginal artery					
		PSV (cm/s)		EDV (cm/s)		RI		PSV (cm/s)		EDV (cm/s)		RI	
		L	R	L	R	L	R	L	R	L	R	L	R
Llama	Fertile	18.20	22.90	3.40	4.70	0.81	0.79	10.90	17.60	5.60	8.20	0.49	0.53
Llama	Fertile	27.90	23.20	6.60	9.30	0.76	0.60	15.90	11.30	10.60	8.60	0.33	0.24
Llama	Fertile	15.10	15.90	4.40	7.70	0.71	0.52	18.10	16.10	7.50	8.50	0.59	0.47
Llama	Fertile	20.80	25.40	5.30	2.00	0.75	0.92	13.20	12.70	8.40	6.90	0.36	0.46
Llama	Fertile	24.80	22.30	7.00	8.50	0.72	0.62	16.10	11.70	8.60	6.70	0.47	0.43
Llama	Fertile	15.40	18.80	1.80	9.70	0.88	0.48	9.80	9.10	5.70	3.90	0.42	0.57
Llama	Fertile	22.70	26.30	8.90	11.90	0.61	0.55	6.40	15.60	3.10	9.80	0.52	0.37
Llama	Infertile	11.10	15.80	3.40	5.40	0.69	0.66	6.30	9.30	4.80	7.80	0.24	0.16
Llama	Infertile	9.40	16.40	3.70	5.80	0.61	0.65	8.50	11.30	5.70	7.80	0.33	0.31
Llama	Infertile	20.40	20.50	4.00	2.50	0.80	0.88	12.30	12.70	6.20	7.60	0.50	0.40
Llama	Infertile	18.20	20.10	7.20	6.60	0.60	0.67	12.60	10.40	7.40	6.30	0.41	0.39
Llama	Infertile	17.30	7.80	5.50	2.80	0.68	0.64	10.00	11.80	6.50	5.90	0.35	0.50
Llama	Infertile	16.00	12.90	2.60	5.10	0.84	0.60	12.30	7.50	8.20	4.90	0.33	0.35
Alpaca	Infertile	12.10	13.30	4.80	3.10	0.60	0.77	9.60	7.80	3.50	3.90	0.64	0.50
